# A General Approach to Achieving Stability and Safe Behavior in Distributed Robotic Architectures

**DOI:** 10.3389/frobt.2018.00108

**Published:** 2018-10-08

**Authors:** Stefan S. Groothuis, Gerrit A. Folkertsma, Stefano Stramigioli

**Affiliations:** ^1^Robotics and Mechatronics Group, Technical Medical Centre, University of Twente, Enschede, Netherlands; ^2^Bio-mechatronics and Energy-Efficient Robotics, ITMO University, St. Petersburg, Russia

**Keywords:** robotics, passivity-based control, energy budgeting, interaction, safety

## Abstract

This paper proposes a unified energy-based modeling and energy-aware control paradigm for robotic systems. The paradigm is inspired by the layered and distributed control system of organisms, and uses the fundamental notion of energy in a system and the energy exchange between systems during interaction. A universal framework that models actuated and interacting robotic systems is proposed, which is used as the basis for energy-based and energy-limited control. The proposed controllers act on certain energy budgets to accomplish a desired task, and decrease performance if a budget has been depleted. These budgets ensure that a maximum amount of energy can be used, to ensure passivity and stability of the system. Experiments show the validity of the approach.

## 1. Introduction

**Figure 1:**
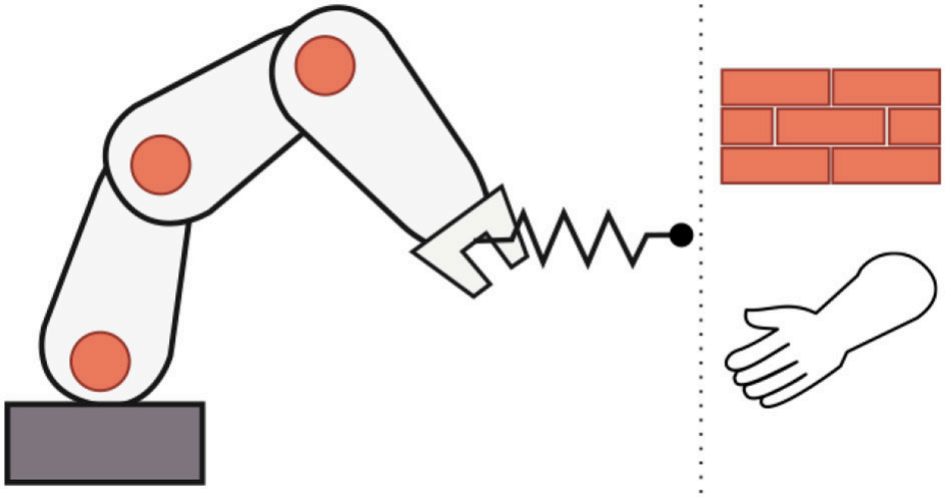
Impedance controlled robot interacting with an environment or a human.

For any controlled robotic system which interacts with an unknown environment, stable interaction, and safety are requirements which cannot be compromised and have to be ensured under all situations. This is even more so for physical human-robot interaction (pHRI), meaning that a robotic system interacts mechanically with a human (Figure 1). An example of a field of application where pHRI is fundamental is in assistive robotics, for instance as developed in the European SoftPro project (Synergy-based Open-source Foundations and Technologies for Prosthetics and RehabilitatiOn). In this project soft synergy-based robotics technologies are developed to design new prostheses, exoskeletons, and assistive devices for upper limb rehabilitation (SoftPro, 2017).

Safety during pHRI is often achieved by limiting performance aspects of the robotic system, e.g., by limiting the maximum velocity or generated force of the system. This may mean that the inherent performance of the robot is decreased since these measures are often implemented in physical ways by, for instance, low power motors and mechanical slip clutches, as opposed to controlled limitations that are bandwidth dependent and, therefore, not strictly safe in all situations (Groothuis et al., 2013). Furthermore, controlled safety measures are often implemented using digital computers, so they act on the physical system in a discrete (sampled) way.

Safety and stability have been addressed by passivity-based control schemes. Passivity means that the stored energy in a system is always less than or equal to the initial amount of stored energy plus the amount that has been added to it (Willems, 1972). In other words: passive systems cannot generate energy themselves. In Schindlbeck and Haddadin (2015), a task-based energy-tank method to obtain passivity was introduced, which cancels out non-passive terms in the system by properly choosing the tank dynamics. At the same time, the tank allows the system to use a certain maximum amount of energy to execute a particular task. This means that during pHRI, a robot can never inject more energy into a human operator or user than what is determined by the controller. This prevents the unbounded growth of system states, and thereby increases safety. Although these continuous-time control laws were successfully implemented on a robotic arm and were proved to be passive, it is important to consider the effects of time discretization, e.g., computation delays, measurement digitization (quantization), and (variable) communication delays or even communication loss between a high-level controller, lower-level actuation controllers, or any other distributed architecture. If, for instance, communication loss results in a failure of torque command updates to any of the low-level controllers, passivity cannot be guaranteed any longer. Time delays are a common problem in telemanipulation and haptic interfaces, where passivity has already been used to stabilize systems subject to time delays. In Lee and Huang (2010), the Passive Set-Position Modulation (PSPM) method was proposed. This method passivates a system by implementing a spring coupling with damping injection between a system's position and its commanded setpoint. The setpoint can be modulated up to what is allowed to keep passivity. This method does require a model of the system. The Passivity Observer/Passivity Controller (PO/PC) as presented in Ryu et al. (2004, 2005) implements a passivity observer, monitoring the energy flowing into and out of a system, while using a passivity controller to dissipate any excess energy, i.e., energy that was not first injected intentionally, that is generated by a system. Experiments were shown, for which a precise measurement of the interaction forces was necessary. It was noted that achieving system passivity may be difficult or impossible due to actuator saturations.

This work proposes a unified energy-based modeling and control paradigm for distributed controlled robotic systems in which passivity for guaranteed stability during pHRI is used, while energy limits are imposed for safety with respect to humans. Passivity is enforced at the actuation layer, i.e., the place in a system where control messages are translated to physical energy flowing into the system. Because it is enforced at the actuation layer, no model of the complete system is necessary. Also, the approach is modular, making the extension of the system straight-forward. The paradigm is based on the fundamental notion of energy in a system, and the energy exchange between systems during interaction. A universal framework that models actuated and interacting robotic systems is proposed, which is used as the basis for energy-based and energy-limited control. This control may be a continuous-time physical (or physically equivalent) controller, or a digitally implemented discrete-time controller. Fundamentally, the controllers are distributed, as opposed to centralized or decentralized, in the sense that decision-making is done not only in the supervisory controller but also in the lower-level actuation controllers. This is analogous to organisms with a central and peripheral nervous system in case of reflex movements for instance. Furthermore, the controllers act on certain energy budgets to accomplish a desired task and take appropriate measures if a budget has been depleted. These budgets ensure that a maximum amount of energy can be used, to ensure passivity of the system. The allocation of the budgets that the controllers may use is proposed. In Schindlbeck and Haddadin (2015), this estimated allocation was done based on the virtual controller energy. Here, also the error energy is included, i.e., the difference between the desired stored energy and the actual stored energy, to more accurately estimate the necessary budget, and which is furthermore divided into individual actuator budgets. A strategy to follow in case a budget has been depleted is presented. With this approach the actuators, that are responsible for energy injection, become “energy-aware,” i.e., they become aware of the amount of energy that is exchanged with a system (Stramigioli, 2015; Folkertsma et al., 2018). The high-level coordinating control loop and the adherence to passivity are separated since passivity is enforced locally in the distributed actuation controllers that are as close as possible to the mechanical system.

The paper is structured as follows: section 2 introduces concepts from port-based modeling, and presents a generic model for a robotic system. In section 3 passivity, and energy-aware systems and actuation are presented. Section 4 presents the energy distributions in the systems, and treats the approach to estimate budget requirements. These requirements are translated to individual actuation budgets that are allocated. Interaction experiments with a setup were performed that are presented in section 5, and the proposed approach presented in this paper and the experimental results are discussed in section 6. The paper concludes with section 7.

## 2. Nature-inspired port-based modeling and control framework

### 2.1. Energetic modeling through interconnections

**Figure 2:**
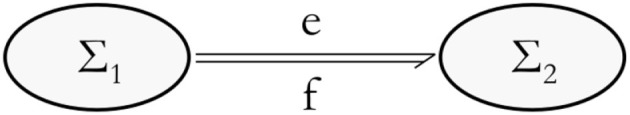
Representation of a power port with a bond (half arrow) indicating a positive power flow from system Σ_1_ to system Σ_2_.

Energy is a fundamental property of all physical systems. A robot is a physical system which follows the laws of nature and can exchange physical energy with the environment (a wall, an object, or a human) through a mechanical interaction. Oliver Heaviside's energy current principle states that if energy goes from one place to another, or from one subsystem to another, it has to travel the space in between, and cannot simply disappear and reappear (Yavetz, 1995). Together with the first principle of thermodynamics, i.e., the energy of an isolated system is constant, it implies that if a system is broken into parts the system can be decomposed energetically in subsystems that exchange energy. An energy increase in one subsystem needs to be accompanied by an energy decrease of the same amount in one or more other subsystem(s). This transport of energy from one subsystem to the other can be modeled by the concept of a power port through which energy can leave one system and enter another, as shown in Figure 2 for systems Σ_1_ and Σ_2_. The corresponding instantaneous energy change is a power flow and can be expressed as a tensor contraction of a variant and a covariant tensor which are called flow *f* and effort *e* (Duindam et al., 2009). In Figure 2, a positive power flow is directed from Σ_1_ to Σ_2_ and is indicated with a half-arrow called a bond. The flows and efforts in the mechanical domain correspond to velocities and forces, respectively^1^. In multibody dynamics, the flows will be twists and are elements of the Lie algebra *T* ∈ *se*(3), and efforts will be wrenches and are the dual elements belonging to the dual Lie algebra *W* ∈ se^*^(3). A power port may then be indicated with (*T, W*).

The port-Hamiltonian formalism for the modeling of systems makes use of this principle (van der Schaft, 2006). The dynamics of any physical system can be modeled in a consistent energetic way by describing it as the interconnection of subsystems that can store energy (generalized energy storage of potential and kinetic energy), reversibly and power continuously transform efforts and flows (transformers, gyrators, and junction structures representing Kirchhoff's laws), and irreversibly transform energy to heat (resistors, dampers). The power continuous connections are composed of elements that together form a Dirac structure, which is a mathematical structure in which no energy is generated or dissipated, but only transformed and distributed (van der Schaft and Cervera, 2002). The Dirac structure determines how the ports are interconnected. Furthermore, this methodology allows to describe open systems, by defining “unconnected” ports which can be used to interconnect the system with another system. An important fundamental feature of this formalism is that the interconnection of systems in this form will again result in a system of the same form, giving rise to a “system algebra.”

### 2.2. The nervous system for robot control

**Figure 3:**
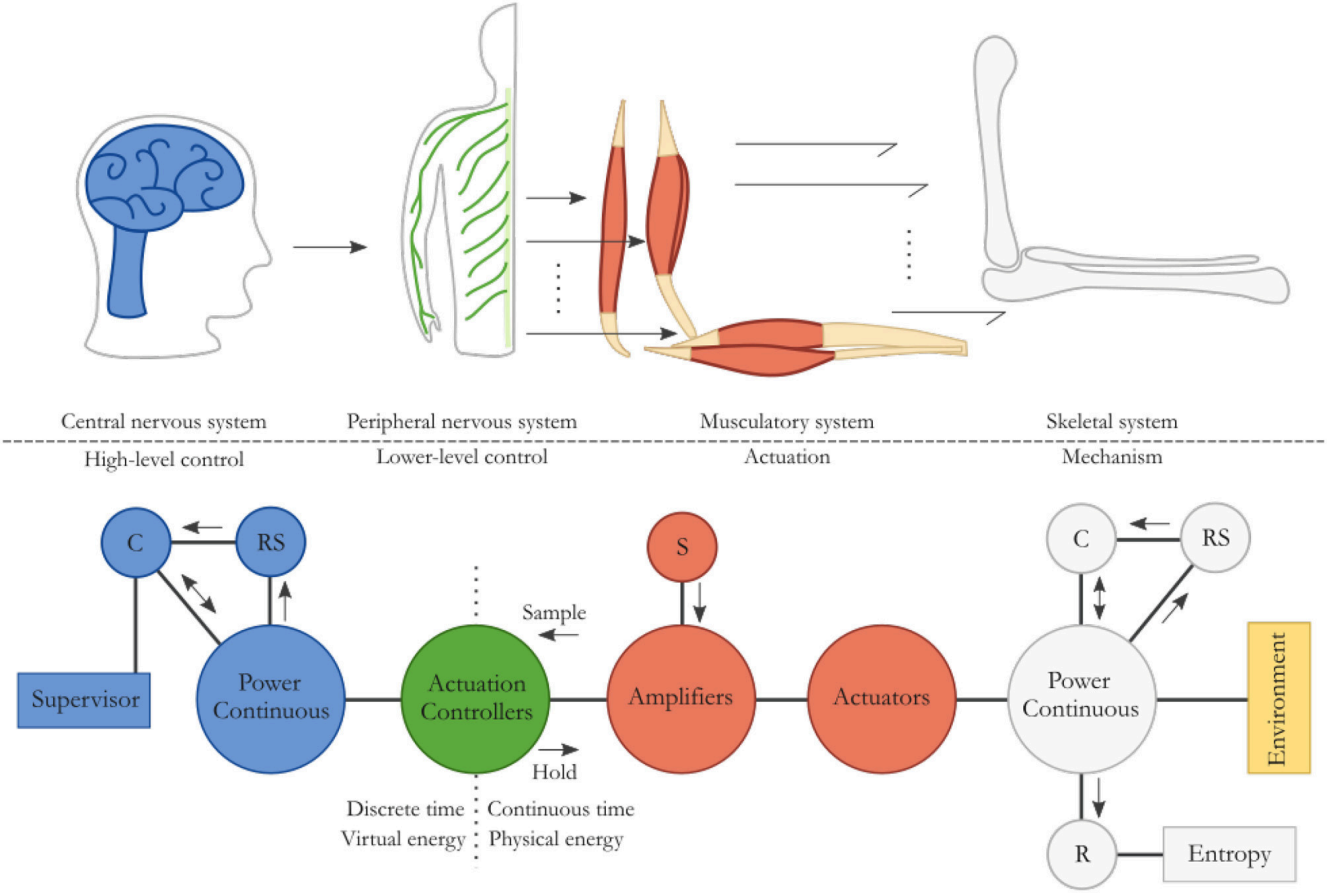
The human motor control system is highly suitable as model for robot modeling and control. The nervous system is responsible for cognition, planning, and controlling actions, while the musculatory system delivers power to generate movement supported by the skeletal system. This corresponds to a high-level and lower-level controller in a robotic system that control actuators to manipulate a mechanism.

Like humans, many organisms regulate their movements using a nervous system. The human motor control system is comprised of several components. The central nervous system (CNS) consists of the brain and spinal cord, and mainly the brain acts as high-level supervisor responsible for cognition and planning. The peripheral nervous system (PNS) consists of the nerves to connect all parts of the body to the CNS, and is responsible for local lower-level control (for instance reflex behavior together with circuits in the spinal cord) and activation of muscles. The musculatory system to move the body is supported by the skeletal system, and the latter defines the kinematic structure and its constraints, i.e., the possible movements of the body. This is a layered or hierarchical approach that can very beneficially be applied to the control of robotic systems. It is the inspiration for the universally applicable model for possibly interacting robotic systems, which is proposed and presented in Figure 3.

Starting from the righthand side in Figure 3, the robotic “skeleton” is the mechanism with a certain kinematic structure, i.e., the load. It is the power continuous interconnection of an energy storage element *C*, associated to the kinetic and potential energy storage of the mechanism. Inherent friction or damping is represented by *R*, which irreversibly transforms energy to heat, increasing the entropy. If additional physical damping is desired to specifically lower the kinetic or potential energy in the system, that energy does not have to be dissipated but can be transformed appropriately and stored in other storage elements. This makes the additional desired damping regenerative, using a transformer indicated with *RS*. This buffered energy can be reintroduced as useful kinetic or potential energy. The system can interact with the environment by an energy exchange, which may cause the energy storage of the mechanical system to change.

The mechanism can be actuated to do useful work and to behave in a desired way. Many actuators, as depicted to the left of the mechanism, for instance electric motors, may power the mechanical system in the same way that many muscles actuate the skeleton. The power amplifiers take their energy needed for actuation from the energy source *S*, which is an “infinite source” within the context of the system or situation.

The power amplifier is controlled by a local actuation controller, like the distributed PNS that locally controls the activation of muscles, as shown to the left of the actuation. This is a relatively fast controller that can act on either high-level commands or on local situations. A reflex arc in the human body, retracting one's hand from a hot surface for instance, is a local control circuit during which an appropriate action is taken without the brain being involved (Purves et al., 2012).

High-level commands can come from the supervisory control system that does the cognition and planning, like the CNS, as depicted on the far lefthand side in Figure 3. It can change a system's behavior by shaping the energy in the system. The energy in storage element *C* can increase if energy needs to be removed from the system, while it may decrease if more energy needs to be provided to the system. Any damping behavior is virtual and does not necessitate the dissipation of physical energy from the system. Instead, it can be regenerative using *RS*, rerouting energy back into a storage element.

This novel philosophy of modeling and controlling a robotic system is similar to the human motor control system. It is beneficial for implementing desired system properties like stability through passivity because of the explicit energetic modeling through interconnections and the layered or hierarchical control system approach.

## 3. Energy-aware systems

Before desired energetic properties can be implemented in the system, it needs to have a way to estimate the energy that was injected or extracted. This section explains the concept of passivity, and presents a method to achieve energy-awareness in robotic systems, which was published previously (Folkertsma et al., 2018) and is summarized here for completeness.

### 3.1. Necessary passivity

In physical systems, the property of passivity is an energy-based measure of stability. It is a special case of dissipativity, as introduced in Willems (1972), that arises naturally in physical dynamical systems. General dissipativity is defined by considering a system

(1) $$\begin{array}{r} ẋ=f\left(x,w\right),\;z=g\left(x,w\right), \end{array}$$

where *x* is the system state, *w* the input, and *z* the output, which take their values in their respective manifolds *X*, *W*, and *Z*. The supply function is a mapping

$$\begin{array}{l} s:W\times Z\mapsto \mathbb{R}. \end{array}$$

The system (1) is said to be dissipative if there exists a storage function *H* : *X* ↦ ℝ such that

(2) $$\begin{array}{r} H\left(x\left(t_{1}\right)\right)\leq H\left(x\left(t_{0}\right)\right)+\int_{t_{0}}^{t_{1}}s\left(w\left(t\right),z\left(t\right)\right)dt \end{array}$$

for *t*_1_ ≥ *t*_0_. The system is conservative, i.e., non-dissipative, if the equality holds in (2). In physical systems, a natural choice for the storage function *H* is the total energy in the system; the supply function is the supplied power of the input port of the system, e.g., the contraction of mechanical force and velocity, i.e., *P* = *F*^T^ẋ in coordinates. Indeed, systems that contain only passive physical elements, i.e., masses, springs, and dampers, can never contain more energy than initially present. Consequently, the energy is bounded and thus a system that is overall passive is always stable (van der Schaft, 1999; Ortega et al., 2008).

If a robot interacts with an environment, the total dynamical system that should be considered is the coupled system of the robot with the environment. This can be represented as two systems coupled by an energy connection that transfers energy from one system to the other. An environment is unknown or very difficult to model adequately, and therefore it is not trivial to ensure that the coupled system is stable if feedback control is used that only considers the model of the system. The environment is not merely a disturbance, but it is part of the system.

When the system with which the controlled robot is interacting is passive, a necessary condition for the stability of the interconnected system is that the controlled robot, as seen from the port (*T, W*), is passive, or in other words: the energy which can be extracted via (*T, W*) is bounded. This is proved in Stramigioli (2015), by constructing a passive environment which would keep on extracting energy from the controlled system in case the controlled system would not be passive. In such a case, the state of the passive environment would diverge, resulting in instability. If the environment with which the controlled robot is interacting is active, the robot should not only be passive, but should be designed in a way that its damping injection is sufficient to dissipate enough energy generated by the active environment.

### 3.2. Discrete energy-aware control and actuation

When considering that the robot should be passive, a sufficient and effective way to achieve the passivity requirement is to use control by interconnection (Stramigioli, 2001). This considers, for example, the control of a system through the connection of parts which may be physically interpretable. Control of a system is then achieved by physically adapting the system by, for instance, attaching springs and dampers, or by any other energy bounded virtual dynamics. This method will not compromise passivity, and, therefore, stability. The classical way of control is to apply forces with actuators which are steered by digital controllers. This method can very likely compromise passivity of the system, because the actuators can possibly inject an unbounded amount of energy into the system. Normally, when using the second method, any state or signal of the robot is measured and an appropriate force or torque *F* is calculated and applied to the system, without considering what the injected power *P* = *F*^*T*^ ẋ would be. This “energy-ignorant” way of control can result in an active system, thereby endangering stability.

An unbounded injection of energy can also occur in case time delays are present in the system. Passivity will be lost due to time delays since an unknown amount of energy may have been injected into or extracted out of the system in between sampling moments. Therefore, the physical energy should be monitored, giving rise to passive sampling (Stramigioli et al., 2005). Two conditions are necessary to estimate the energy, which are:

1. a Zero Order Hold (ZOH) should be used to keep the effort during a sampling period constant, and;
2. a configuration sensor should be collocated with the effort.

The energy transfer Δ*H*(*k*) through a generic power port during a time step *T* is given by the integral of the power:

$$\begin{array}{l} \Delta H(k)=\int_{kT}^{(k+1)T}e_{d}^{T}(k)f(s)ds\\ \;=e_{d}^{T}(k)\int_{kT}^{(k+1)T}f(s)ds\\ \;=e_{d}^{T}(k)(x((k+1)T)-x(kT)). \end{array}$$

A slight adaptation can be applied to obtain a computable form:

(3) $$\begin{array}{r} \Delta H\left(k-1\right)=e_{d}^{\text{T}}\left(k-1\right)\left(x\left(kT\right)-x\left(\left(k-1\right)T\right)\right). \end{array}$$

The effort is held constant using a ZOH, while the flow is a continuous time physical variable that is sampled. If this energy sampling concept is applied in an actuator, that actuator becomes aware of the energy it injects or extracts. This type of actuator is the Embedded Energy-Aware Actuator, or E^2^A^2^ (Folkertsma et al., 2018). Using this type of actuator ensures passive behavior from a control perspective at the interface of the signal and energy domains, i.e., at the actuator. The actuator takes its energy from the source *S* in Figure 3 to perform work, while that energy is monitored as virtual energy according to (3).

### 3.3. Beyond passivity: safe interaction

Passivity ensures that never more energy can be extracted from a system than what has been added to it previously. This, however, does not entail safety with respect to humans during pHRI. Extracting a certain limited amount of energy present in a system is a passive interaction, but that limited energy may be delivered in such a way that human injury can occur. The amount and rate of energy exchanged should be within safe levels, which is the maximum energy that can be transferred during interaction without causing injury. This amount is based on a criterion like the Maximum Power Index (Newman et al., 2000; Alami et al., 2006), which is an approximation of the Wayne State Concussion Tolerance (Greenwald et al., 2008). Injury to the head is quantified as maximum power that can be transferred, which over time is transferred energy. Considering a robot that may move and interact, this limits its kinetic energy within safe levels as set a priori. Previous work has presented a way to let the system adhere to energy limits by decreasing elasticity or damping to limit the potential or kinetic energy, respectively (Tadele et al., 2014). This has been shown to be applicable to higher dimensional systems that are controlled by 6D spatial springs as well (Raiola et al., 2018).

## 4. Distributed energy and budgeting

### 4.1. Physical and virtual energy storage

Figure 3 shows the model of an energy-based framework that can be used to describe the modeling and control of generic robotic systems. Energy is stored in several places, and the distinction can be made between virtual and physical energy. Physical energy is associated to the mechanism and actuation, and is stored as kinetic and potential energy in the robot. Virtual energy storage is associated to the supervisory controller and the lower-level actuation controllers, and, if implemented digitally, only exists as numbers in software. For the robot to perform desired tasks, and to do mechanical work, it needs a certain amount of energy. This energy may be present as stored energy in the mechanism, or should be injected through the environment or the actuation. To ensure a passive system, the amount of energy that the actuators inject is monitored by motor controllers through the use of the E^2^A^2^ actuators. A virtual energy budget, representing the physical energy that may be injected by an actuator, is defined, and if that budget is depleted no further physical energy is allowed to be injected in the system by that actuator. The supervisor has an energy budget to distribute among the individual actuators based on the high-level control implementation. When the controllers are implemented digitally, budgeting is done in a discrete way, allocating energy each time step.

Due to the distributed nature of the system, the supervisor will likely be implemented on a different system than the actuation controllers. That means that the sampling frequency at which they operate may be different. It is assumed in the remainder that the supervisor has a sampling time of *T*_*k*_, with sampling moments *k*, while the actuation controllers have a sampling time of *T*_*n*_*j*__, with sampling moments *n*_*j*_ for actuation controller *j*. Furthermore, it holds in general that *T*_*k*_ > *T*_*n*_*j*__ ∀*j*, i.e., the supervisor is slower than the actuation controllers.

### 4.2. Energy requirements

A fundamental question that arises is: “How can the various budgets be determined such that stability is guaranteed and system performance is not limited by conservative budgeting?.”

One way to answer this question is to use a teaching approach. A robot can be externally manipulated and thereby “shown” a certain motion, which it uses to observe the evolvement of system states. These states are directly related to the kinetic and potential energy (changes) in the system, that will have to be injected by actuators, and are, hence, the actuation controller budget requirements. However, it might be cumbersome or even impossible to teach a robot a certain motion, and this seems only useful for repetitive tasks and motions. Therefore, a model-based approach is proposed here. A model of the robot is very likely developed for designing a controller, which can be directly used for estimating energy requirements during motions.

Consider a generic dynamic model of a robot in joint coordinates *q*, i.e.,:

(4) $$\begin{array}{r} M\left(q\right)\ddot{q}+C\left(q,\dot{q}\right)\dot{q}+B\dot{q}+g\left(q\right)=\tau +\tau_{ext}, \end{array}$$

where *M*(*q*) is the inertia matrix of the robot, $C\left(q,\dot{q}\right)$ is the matrix associated with Coriolis and centrifugal forces, *B* is the joint damping matrix, *g*(*q*) are the configuration dependent potential forces, τ are the controlled joint torques, and τ_*ext*_ are other external forces acting on the joints.

A certain motion task that is to be executed by the robot requires an amount of energy to be converted into kinetic energy. An accelerated motion will always correspond to a change of kinetic energy *E*_*kin*_, and if a system moves along a gravitational field, for instance increasing and decreasing its height, the potential energy *E*_*pot*_ will change as well. Some energy is dissipated as heat and, thereby, irreversibly removed from the system. These losses are due to friction, for instance. These energies are bounded in case of servoing tasks like position setpoint regulation from an initial condition. Therefore, the energy requirement to accomplish such tasks can be found in a straight-forward way. However, in case of periodic motions that may continue indefinitely, the energy requirement cannot be given as one energy budget that should suffice for accomplishing the task, since the required energy of the dissipative system will increase to infinity as the periodic motion execution time tends to infinity. An energy requirement for a motion during a certain time window can, however, be given. This means that for a finite time window, the energy requirement is also finite. Therefore, energy budget allocations for generic tasks and movements can only usefully be done for finite time windows. Since controllers are mainly implemented in a discrete way in computers running at a certain sampling interval, it makes sense to consider the energy requirements during each time step and providing an energy budget suitable for that time step.

In Schindlbeck and Haddadin (2015), the accurate tracking of a desired contact force was considered for which the required energy budget was estimated based only on the virtual energy in the impedance controller. A quadratic potential energy function in work space was defined, with which the energy budget was initialized. Here, the energy allocation for a time step is based on the virtual energy present in the controller, and on the kinetic and potential energy errors, i.e., the difference between the energies associated to desired system states and the actual system states. Furthermore, this energy is divided into the individual actuator controller budgets, and will be allocated as such.

#### 4.2.1. Impedance controller

Suppose a robot's end-effector is controlled to track a desired trajectory (*r*_*d*_, ṙ_*d*_) in work space. A generic impedance controller can be given by:

(5) $$\begin{array}{r} F_{imp}:=-K_{r}\tilde{r}-B_{r}ṙ, \end{array}$$

in which *F*_*imp*_ is the virtually applied work space force to the end-effector, *K*_*r*_ is the work space elasticity matrix, $\tilde{r}=r-r_{d}$ is the deviation between the actual and desired end-effector work space configurations, and *B*_*r*_ is the work space damping matrix. The joint coordinates can be transformed to work space coordinates using a Jacobian mapping, as given by:

(6) $$\begin{array}{r} ṙ=J_{r}\left(q\right)\dot{q}, \end{array}$$

in which *J*_*r*_(*q*) is the configuration dependent Jacobian matrix mapping joint velocities $\dot{q}$ to end-effector velocities ṙ. The dual relation maps end-effector forces to joint forces or torques by:

(7) $$\begin{array}{r} \tau =J_{r}^{\text{T}}\left(q\right)F, \end{array}$$

in which *F* is the end-effector force in work space. With (7) the impedance controller of (5) can be expressed as joint space torques by:

(8) $$\begin{array}{r} \tau_{imp}=J_{r}^{\text{T}}\left(q\right)F_{imp}=-J_{r}^{\text{T}}\left(q\right)\left(K_{r}\tilde{r}+B_{r}ṙ\right), \end{array}$$

in which τ_*imp*_ is the controlled end-effector force expressed as joint torques.

#### 4.2.2. Virtual controller energy

The mapping in (6) relates velocities, but, equivalently, infinitesimal displacements, and displacement errors, can be related by:

$$\begin{array}{l} \delta r=J_{r}\left(q\right)\delta q, \end{array}$$

(9) $$\begin{array}{r} \delta \tilde{r}=J_{r}\left(q\right)\delta \tilde{q}. \end{array}$$

The change in stored controller energy due to a small displacement away from the desired (equilibrium) configuration can be given by:

$$\begin{array}{l} E_{contr}:=\frac{1}{2}\delta {\tilde{r}}^{\text{T}}K_{r}\delta \tilde{r}, \end{array}$$

which, together with (9), can be expressed as a function of the joint configuration by:

$$\begin{array}{l} E_{contr}=\frac{1}{2}\delta {\tilde{q}}^{\text{T}}J_{r}^{\text{T}}\left(q\right)K_{r}J_{r}\left(q\right)\delta \tilde{q}, \end{array}$$

which can be written as:

$$\begin{array}{l} E_{contr}=\frac{1}{2}\delta {\tilde{q}}^{\text{T}}K_{r}^{q}\delta \tilde{q}, \end{array}$$

where $K_{r}^{q}$ is the end-effector controller elasticity expressed in joint space, i.e., the pull back of *K*_*r*_, given by:

$$\begin{array}{l} K_{r}^{q}:=J_{r}^{\text{T}}\left(q\right)K_{r}J_{r}\left(q\right). \end{array}$$

#### 4.2.3. Energy stored in mechanism

Besides this controller energy, the energy in the mechanism may deviate from the energy that would be present in case the system states are as desired. That means that the total energy deviation can be given by:

(10) $$\begin{array}{r} {Ẽ}_{tot}:={Ẽ}_{kin}+{Ẽ}_{pot}+E_{contr}, \end{array}$$

which is the energy that the actuation can still add to the system, and in which the kinetic and potential energy errors are defined as:

(11) $$\begin{array}{r} {Ẽ}_{kin}:=E_{kin_{d}}-E_{kin}, \end{array}$$

(12) $$\begin{array}{r} {Ẽ}_{pot}:=E_{pot_{d}}-E_{pot}. \end{array}$$

The kinetic and potential energies of the robot can be given by:

$$\begin{array}{ll} E_{kin} & :=\frac{1}{2}{\dot{q}}^{\text{T}}M\left(q\right)\dot{q},\\ E_{pot} & :=\overset{I}{\underset{i=1}{\sum }}m_{i}gh_{i}, \end{array}$$

while the corresponding desired energies are defined by:

(13) $$\begin{array}{r} E_{kin_{d}}:=\frac{1}{2}{\dot{q}}_{d}^{\text{T}}M\left(q_{d}\right){\dot{q}}_{d}, \end{array}$$

(14) $$\begin{array}{r} E_{pot_{d}}:=\overset{I}{\underset{i=1}{\sum }}m_{i}g{h_{d}}_{i}. \end{array}$$

Hence, the (desired) potential energy is defined by the (desired) center of mass height *h*_*i*_ and mass *m*_*i*_ of each individual body. It may be assumed that the inertia matrix in the desired configuration *M*(*q*_*d*_) is approximated by the inertia matrix in the actual configuration *M*(*q*), which is valid in nominal situations in which the time step is relatively small compared to the system dynamics.

#### 4.2.4. Individual actuator budgeting

Equation (10) is the total energy deviation of the robot as expressed in joint coordinates. For a distributed system, individual actuator budgets should be derived, so that each actuator explicitly adheres to the passivity requirements. Therefore, the energies derived before are separated to isolate individual actuator contributions:

$$\begin{array}{l} {\vec{E}}_{contr}:=\frac{1}{2}\text{diag}\left(\delta \tilde{q}\right)K_{r}^{q}\delta \tilde{q}, \end{array}$$

in which diag(…) is an operator transforming a vector into a diagonal matrix, and ${\vec{E}}_{contr}$ is an array of individual actuator energy contributions. Note that the sum over all elements of array ${\vec{E}}_{contr}$ is the scalar *E*_*contr*_. The same is done with the kinetic energy:

$$\begin{array}{l} {\vec{E}}_{kin}:=\frac{1}{2}\text{diag}\left(\dot{q}\right)M\left(q\right)\dot{q}, \end{array}$$

and the potential energy:

$$\begin{array}{l} {\vec{E}}_{pot}:=\text{vec}\left(m_{i}gh_{i}\right), \end{array}$$

where vec(…) is an operator that creates an array from scalar elements *i*, where *i* identifies a mechanism body. The same is done with (13) and (14) for the desired energies.

The energy deviations of the actuation controllers can now be given in an array as the sum of the various energy contributions:

$$\begin{array}{l} {\vec{\tilde{E}}}_{tot}:\text{​​}={\left[\text{​​}\begin{array}{cccc} {\tilde{E}}_{1}, & {\tilde{E}}_{2}, & \ldots , & {\tilde{E}}_{N} \end{array}\text{​​}\right]}^{\text{T}}\\ \;={\vec{\tilde{E}}}_{kin}+{\vec{\tilde{E}}}_{pot}+{\vec{E}}_{contr}, \end{array}$$

in which Ẽ_*j*_ is the energy deviation associated to actuation controller *j*, and ${\vec{Ẽ}}_{kin}$ and ${\vec{Ẽ}}_{pot}$ are the array forms of (11) and (12).

### 4.3. Energy budget allocation

Each supervisor time step *k* the actuation controller budgets are replenished up to an energy level that is calculated using the error energy. The new energy setpoint at time *k* = *k*_*s*_ for controller *j* is indicated with *E*_*s*_*j*__(*k*), which is sampling moment *n*_*k*_*s*__ for the actuation controllers.

Defining *E*_*b*_*j*__ as the energy budget of controller *j*, $E_{b_{j}}^{-}=E_{b_{j}}\left(n_{k_{s}}-1\right)$, and ϵ as some small amount of energy which is explained in section 4.4, the allocation at time *k* can be defined as:

(15) $$E_{s_{j}}(k):\text{​​}=\{\begin{array}{ll} {\tilde{E}}_{j}(k-1)\geq 0: & {\tilde{E}}_{j}(k-1)\\ {\tilde{E}}_{j}(k-1)<0: & \{\begin{array}{ll} E_{b_{j}}^{-}>0: & E_{\epsilon }\\ E_{b_{j}}^{-}\leq 0: & \{\begin{array}{ll} E_{b_{j}}^{-}>{\tilde{E}}_{j}(k): & E_{b_{j}}^{-}\\ E_{b_{j}}^{-}\leq {\tilde{E}}_{j}(k): & {\tilde{E}}_{j}(k) \end{array} \end{array} \end{array}$$

Hence, a controller budget *E*_*b*_*j*__ can maximally increase up to the error energy Ẽ_*j*_. If more energy is still in the actuation controller budgets than what is necessary for overcoming the deviation in the states, no additional energy is needed and energy may even be removed. Excess energy will flow back to the supervisor to be distributed in future time steps. When the appropriate energy setpoint has been determined, the corresponding budget level is set to this setpoint, i.e., *E*_*b*_*j*__(*n*_*k*_*s*__) = *E*_*s*_*j*__(*k*).

This means that the energy budget setpoint is an absolute energy level that resets a local controller budget to the setpoint, as opposed to a relative energy level causing an increase or decrease of the current local controller budget. The former method prevents drift-like issues, e.g., unnecessary virtual energy dissipation if energy budget messages are lost in the communication network.

It is assumed that the actuation controllers operate at a higher frequency than the supervisor. This means that the energy budget that is allocated is the permissible energy to be used during multiple time steps *n* until a new budget is allocated at supervisor time step *k*+1. The energy that has been used during an actuation controller time step is:

$$\begin{array}{l} E_{used_{j}}\left(n-1\right):=\tau \left(n-1\right)\cdot \left(q\left(n\right)-q\left(n-1\right)\right), \end{array}$$

which is the computable form as given in (3). The energy used since the energy budget was updated by a new setpoint at time *n*_*k*_*s*__ < *n* is:

$$\begin{array}{l} {Ē}_{used_{j}}\left(n\right)={Ē}_{used_{j}}\left(n-1\right)+E_{used_{j}}\left(n-1\right), \end{array}$$

and the energy left in the budget of an actuation controller is then:

(16) $$\begin{array}{l} E_{b_{j}}\left(n\right)=E_{s_{j}}\left(k\right)-{Ē}_{used_{j}}\left(n\right).\text{(18)} \end{array}$$

With this implementation of the E^2^A^2^, a control message communicated over a system bus is no longer just a setpoint ς_*j*_ for torque, velocity, or position, but also includes the energy *E*_*s*_*j*__ as the new energy budget that may be used: σ_*j*_: = (ς_*j*_, *E*_*s*_*j*__). *E*_*s*_*j*__ replaces the local energy budget *E*_*b*_*j*__, while ς_*j*_ is executed until the next setpoint σ_*j*_ is received, or until the energy budget *E*_*b*_*j*__ has been depleted.

### 4.4. Energy budget usage

**Figure 4:**
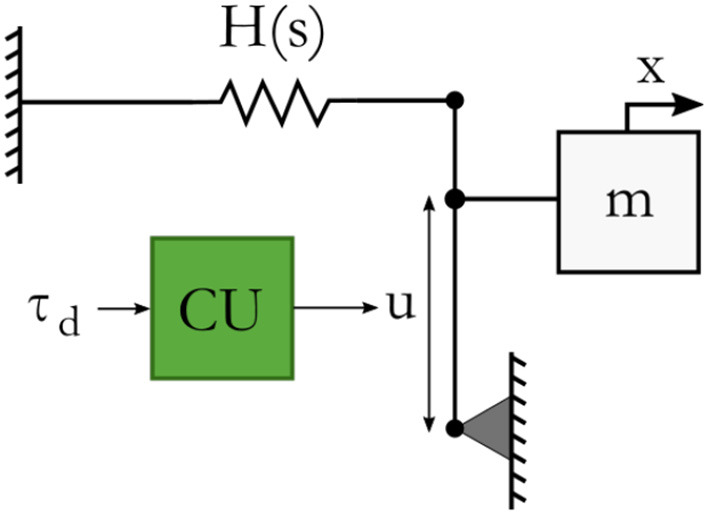
Physical representation of an energy budgeted actuation controller.

The impedance controller of (5) is realized with joint torque control, which means that the setpoints σ will be of the form σ: = (τ, *E*_*s*_). A physical way of representing a force controller on joint level that controls τ and that only has a limited energy budget that can be expended to perform a task, is shown in Figure 4. This system is based on an energy storage element with state *s* and stored energy *H*(*s*), that is coupled to a transformer with transformation ratio *u* as set by a computational unit (CU). The transformer determines how the state of the storage element is transformed to a controlled force τ applied to the mass *m*. In case of a unit storage element the stored energy as a function of the state is defined by:

$$\begin{array}{l} H\left(s\right):=\frac{1}{2}s^{2}. \end{array}$$

The effort *e* generated by the storage element is a force given by:

$$\begin{array}{l} e:=\frac{\text{d}H\left(s\right)}{\text{d}s}=s. \end{array}$$

The force τ on the mass *m* is given by:

$$\begin{array}{l} \tau =u\cdot e, \end{array}$$

So to apply a desired force τ_*d*_ on the mass, the computational unit will calculate a transformation ratio by:

$$\begin{array}{l} u:=\frac{\tau_{d}}{s}, \end{array}$$

such that

(17) $$\begin{array}{r} \tau =u\cdot e=\frac{\tau_{d}}{s}\cdot e=\frac{\tau_{d}}{s}\cdot s=\tau_{d}. \end{array}$$

If the energy budget has depleted, no more energy may be injected in the system by the actuator, so the power flow *P* out of the actuator may not be positive, since that would further inject energy in the system. However, it may be negative as that will extract energy from the connected system. Therefore, the actuation controller should be parameterized as a function of the budget's energy content (Folkertsma et al., 2018; Raiola et al., 2018). More precisely, the calculation of the transformation ratio is parameterized as follows:

(18) $$\begin{array}{ll} u & =\{\begin{array}{ll} \frac{\tau_{d}}{s} & \text{if }((H(s)>\epsilon )\vee (P<0)),\\ \frac{\tau_{d}}{\gamma^{2}}s & \text{if }0<H(s)\leq \epsilon ,\\ 0 & \text{otherwise}. \end{array} \end{array}$$

Here, $\gamma =\sqrt{2\;\epsilon }$, and ϵ is some small amount of energy. If enough energy is in the storage element, i.e., *H*(*s*) > ϵ, *u* can be such that τ = τ_*d*_ according to (17). This also holds if the power is directed such that energy is extracted from the system instead of injected (*P* < 0). If the energy has decreased below ϵ, the force that will be applied is proportional to the energy content. This decreases the rate at which the energy budget depletes (Raiola et al., 2018). In discrete time, in which the physical storage *H*(*s*) will be a discrete calculated budget *E*_*b*_, there is no way to assure that the energy budget will never become negative, because of the discrete sampling of a continuous time system and the corresponding time delays. Therefore, the condition that *H*(*s*) < 0 is included, which means that *u* = 0.

The minimum to which the calculated energy can become negative is $E_{b_{min}}:=\int_{n\cdot T_{n}}^{\left(n+1\right)\cdot T_{n}}P\left(t\right)dt$. Since this is always a finite and generally small amount, it does not compromise stability if it is properly taken into account by subtracting the amount of negative energy from a new budget allocation setpoint.

### 4.5. Energy budget depletion

Energy in the actuation controllers is allocated from the supervisor budget, and, therefore, that budget will decrease by the same amount. It will further decrease due to dissipation in the system, which should be replenished to prevent the supervisor budget from completely depleting. If that happens, the system is unable to perform any action if no energy is added in the supervisor or to the mechanism through interaction. It may also be that not enough energy is allocated to an actuator during a time window, due to model inaccuracies for instance, which results in the system not being able to accomplish a required motion to satisfy a certain task. Depending on the application an actuation controller transmission ratio of 0, meaning an actuator force of 0, may not be desirable. Supposing only one actuator, the energy contents of the system are then not changed by actuation, which means that kinetic energy can only decrease by dissipation, i.e., a motion is not braked but only damped. Possible solutions are provided here.

#### 4.5.1. Braking the system

Whenever, a local energy budget is depleted the system may be braked by removing energy. This can be achieved by engaging a local P-controller that controls a force in the opposite direction of the movement. In that way deceleration is achieved while regenerating kinetic energy as energy in the local motor controller budget, since $P=\tau \cdot \dot{q}<0$.

#### 4.5.2. Exchanging energy

Local actuation controllers may be given the ability to communicate with each other to exchange (parts of) energy budgets. If one controller doesn't need to use its complete budget while another needs additional energy, energy can be shifted to the controller with the depleted budget. This does not alter the total energy contents of the system and, therefore, does not compromise passivity.

#### 4.5.3. Self-replenishing local budgets

In a strict application of the passivity requirement through energy awareness, having insufficient energy can be considered as the inherent safety of the implementation. When a looser approach is followed, while notably still requiring unconditional stability, additional energy may be supplied to the particular budget that has been depleted. This can be done by the local controller by replenishing the energy budget with an amount of energy that is strictly less than the previously allocated energy budget. If this is recursively done, the amount of additional energy will always be finite. It is important that the self-replenished energy is communicated to the supervisor, such that it can keep track of the total energy in the system and decrease its own budget with the same amount to keep a consistent, and passive, total energy level.

The actuation controller's energy budget is given by (16). The additional energy that may be generated at time step *n*, *E*_*b*_*add*__(*n*), is proposed to be:

(19) $$\begin{array}{r} E_{b_{add}}\left(n\right):=\frac{E_{s}\left(k\right)}{a^{l}} \end{array}$$

in which *a* > 1 is a factor that determines what portion of the previously allocated energy is allocated again, and *l* ∈ [1, *L*] is a counter keeping track of the number of times energy was added. *L* is given by the amount of time steps *n* in each (slower) time step *k*, which under communication loss can mean that *L* → ∞. This would mean that the total energy added is:

$$\begin{array}{l} E_{b_{add}}:=\underset{L\to \infty }{\lim}\overset{L}{\underset{l=1}{\sum }}\frac{E_{s}\left(k\right)}{a^{l}}=\frac{E_{s}\left(k\right)}{a-1}. \end{array}$$

For *a* > 1, the additional energy that may be used is *E*_*b*_*add*__ < ∞, while for *a* = 2 it becomes *E*_*b*_*add*__ = *E*_*s*_(*k*).

### 4.6. Complete system

**Figure 5:**
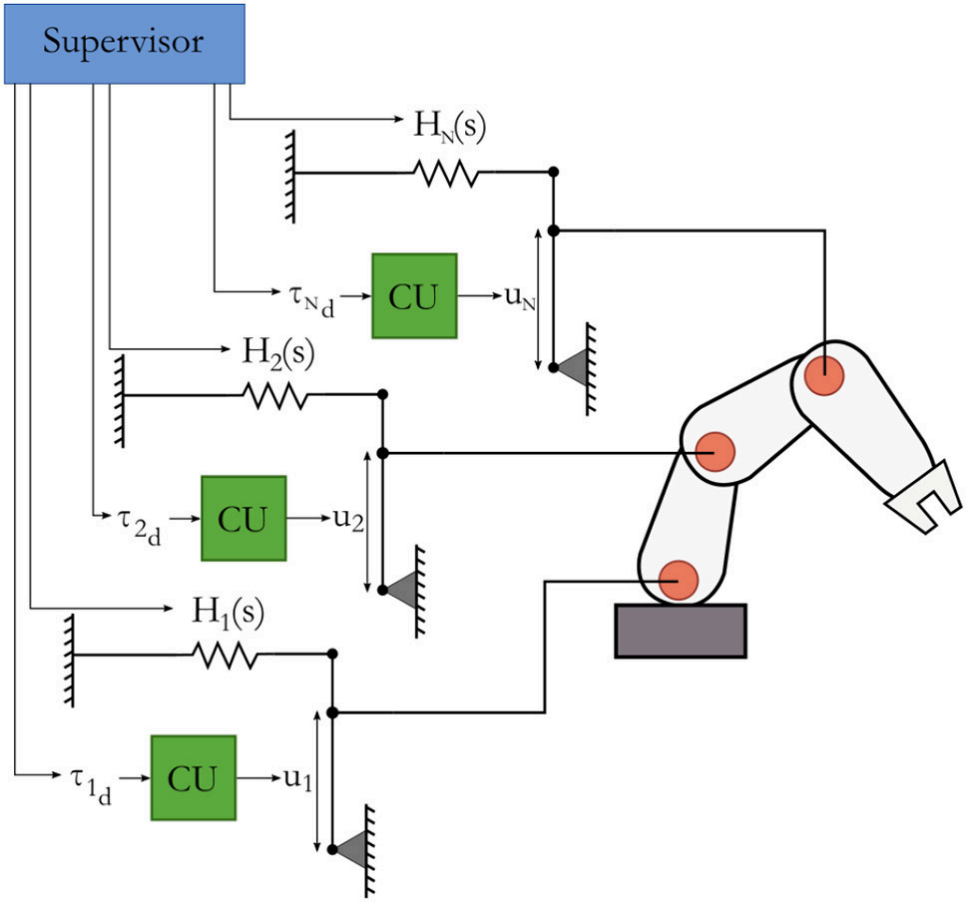
Physical representation of the proposed distributed energy-aware system. The system is structured similarly as the generic model given in Figure 3.

A physical representation of the complete system is shown in Figure 5, which resembles the model as shown in Figure 3. It consists of actuation controllers as the one in Figure 4 that attach to the actuators in the joints of a robot. A supervisor connects to the actuation controllers, and allocates energies in the individual controllers and sets force setpoints in the computational units. These local controllers are energy-aware and adhere to passivity requirements, which means that the mechanism's stability is guaranteed. Time delays or even communication loss between the supervisor and the actuation controllers do not compromise that guarantee.

## 5. Case study

**Figure 6:**
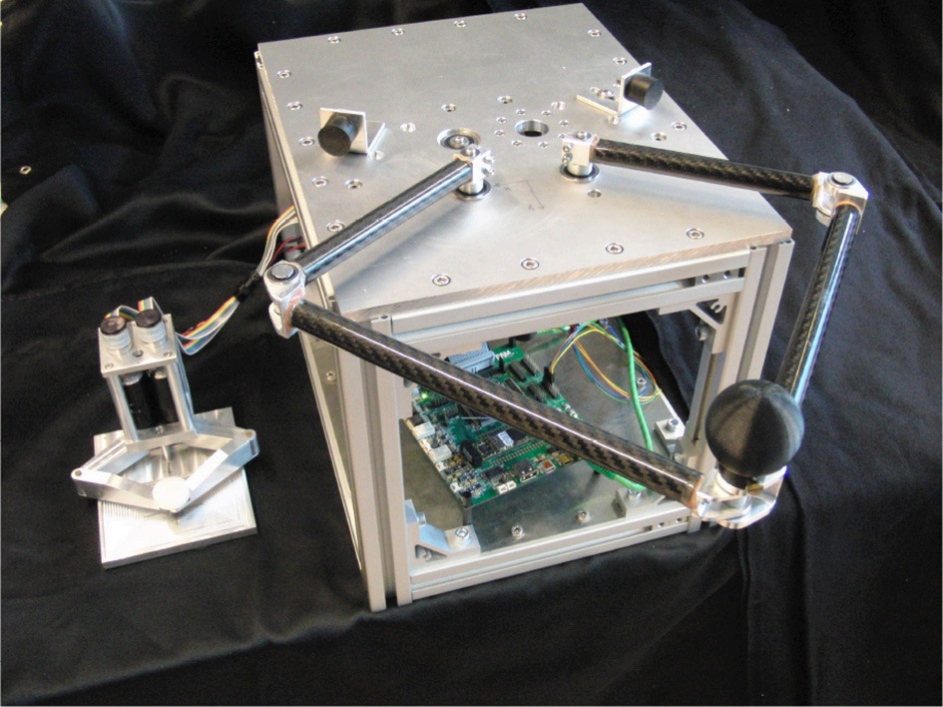
Two coupled five-bar linkage systems are used as the experimentation setup. The smaller system on the left is termed the master while the larger system on the right is the slave.

Experiments were performed to validate the proposed modeling and energy-aware control. This was done with a five-bar linkage system capable of end-effector movements in the horizontal plane. It uses two motors *M*_1_ and *M*_2_ to drive four bars in the horizontal plane. Of the four joints in the setup two are dependent due to the kinematic constraints of the linkage. Therefore, there are two actuated degrees of freedom to control two configuration variables of the end effector in the plane: *x* and *y* position, or either one of the positions *x* or *y* and the orientation. This system may be used as a slave system that is capable of versatile movements and opposing forces. This system is coupled to a similar, but smaller-scale, five-bar linkage system, both shown in Figure 6.

### 5.1. Implementation

The slave system consists of two Faulhaber 3890-048CR DC-motors that drive two links of 15.3 cm in length via a capstan transmission with a speed reduction factor of 7.3. Due to this transmission and low transmission ratio, the actuators are backdrivable. Two other links of 23.8 cm in length are connected to both driven links, and are coupled at the end effector to form a parallel mechanism. The rotation of the motors is measured with optical motor shaft encoders. The two motors are driven by ESCON 50/5 motor controllers in current controlling mode. The master system is similar to the slave system, using two Maxon RE25 DC-motors, each driving a link of 6.3 cm without a gear transmission, to which links of 7.5 cm are connected and coupled at the end effector to form a parallel mechanism. Again, motor rotation is measured using optical motor shaft encoders. The two motors can be driven by ESCON 24/2 motor controllers.

A kinematics and dynamics model of the two systems in the setup were developed. The forward kinematics model is based on straight-forward planar geometry which treats the two “legs” of the systems as 2-DOF planar serial mechanisms, which are coupled by the constraint that the end effectors of both “legs” should be in the same point in space. The end effector position of the parallel mechanism *R* can then also be expressed as the angles of both motors (*q*_1_, *q*_2_) after the transmissions, i.e., $R\left(q_{1},q_{2}\right):={\left[R_{x}\left(q_{1},q_{2}\right),R_{y}\left(q_{1},q_{2}\right)\right]}^{\text{T}}$. The analytical Jacobian *J* is then given by the partial derivatives of *R*(*q*_1_, *q*_2_) with respect to *q*_1_ and *q*_2_. A physical system model of both the master and the slave setups has been developed in the bond graph modeling language with the modeling software package 20-sim (Controllab Products B.V., 2016). Energy budget calculations were done using the Euler-Lagrange model of (4).

The controller as presented in section 4 was included in the setup model. Using the 20-sim 4C package (Controllab Products B.V., 2016), realtime C-code was generated of the controller submodel which was compiled for a Gumstix embedded Linux computer. This Gumstix is incorporated on a RAMstix board that provides inputs and outputs for, for instance, encoders and motor controller PWM signals (Robotics and Mechatronics, 2018). This way, simulated behavior with a model and designed controller is directly implemented on a hardware setup for experimentation. The controller that was implemented is a combination of the supervisor controller and the actuation controllers as presented in Figure 3. In a distributed system the actuation controllers are in general separate controllers accepting setpoint commands to steer the power amplifiers. Here, they are implemented on the same embedded computer as the supervisor for ease of implementation, but can be run at a different sampling frequency than the one set for the supervisor. This can therefore emulate a fast motor controller communicating with a slower supervisor.

### 5.2. Experimental method

A Cartesian planar impedance controller is implemented on the slave system, as given in (5). The master system can dictate the virtual equilibrium point in space of the slave *r*_*d*_ by a transformation *Z* between the master and slave position workspaces *S*_*m*_ and *S*_*s*_:

(20) $$\begin{array}{r} Z:=S_{m}\mapsto S_{s} \end{array}$$

in which subscript *m* and *s* indicate the master and slave system, respectively. If *r*_*m*_ ∈ *S*_*m*_ and *r*_*s*_ ∈ *S*_*s*_, the affine workspaces transformation is defined as:

(21) $$\begin{array}{r} r_{d}=\alpha \left(r_{m}-r_{m_{0}}\right)+r_{s_{0}}, \end{array}$$

in which α is a scalar parameter isotropically scaling the workspace, and *r*_*m*_0__ and *r*_*s*_0__ are the initial positions of the master and slave end effectors, respectively.

This master and slave setup lends itself for the application of repetitive motion rehabilitation given to a patient (the slave operator) by a therapist (the master operator). During experimentation, the slave system is used to perform a back-and-forth motion with an arm. The master operator may support the person by slightly preceding the person's intended movement and thereby causing the impedance controller to pull the person's arm along. The master operator may also obstruct the person by opposing the intended motion causing the person to push against the impedance controller or even completely deviating from the path, to train motion accuracy. Specifically, a back-and-forth motion was performed with the slave in the master's initial end effector position, and after a while the master position is moved to obstruct the slave operator. Furthermore, the virtual spring's equilibrium point of the impedance controller, as set by the master system, is tracked in free space, and the slave system is manipulated (to charge the virtual impedance controller energy) and then released to assess whether instability occurs. These experiments are performed with the energy-based control paradigm as presented, as well as with a traditional controller that is unaware of injected energy, both implemented in discrete time.

Unfortunately, it was observed that the slave system has a relatively high stiction and friction that are position dependent, which is most likely due to bearing misalignement and highly tensioned tendons. Therefore, free space tracking of a virtual spring equilibrium point is relatively inaccurate and jerky. No immediate changes could be made to the setup to solve this issue.

### 5.3. Experiments

The experiments were performed with an impedance controller, which in general is a virtual spring and damper. In this case, however, due to the high friction setup the impedance controller was implemented as only an isotropic spring with elasticity of 50 N/m. The workspace scaling parameter was set at α = 2.

#### 5.3.1. Understanding the energy-based controller

**Figure 7:**
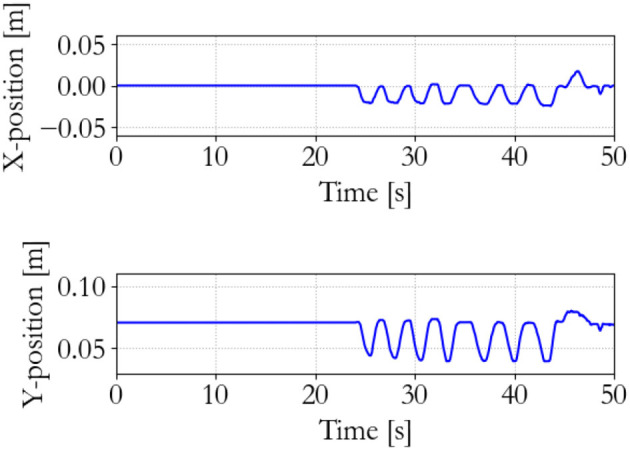
Master end effector positions.

**Figure 8:**
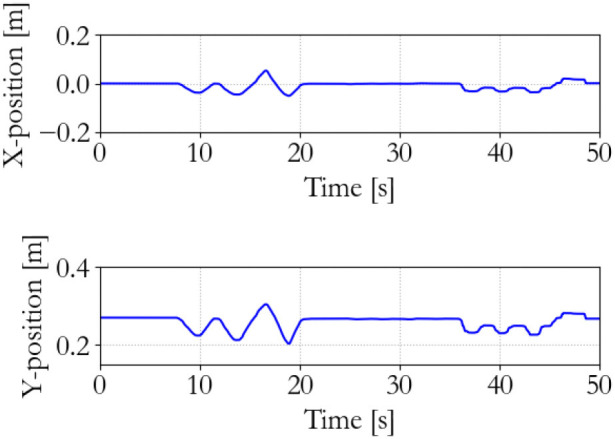
Slave end effector positions.

**Figure 9:**
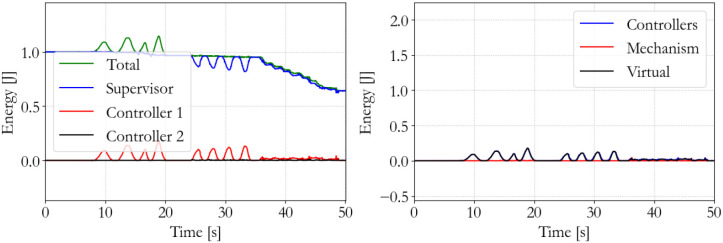
Slave energy levels.

To show the workings of the energy-based control paradigm, an identifying experiment was done at 100 Hz with an initial supervisor energy budget of 1 J, which may be used to inject energy in the system via the motor controllers. The end effector position of the master is shown in Figure 7, the end effector position of the slave is shown in Figure 8, and the energy levels associated to the slave are shown in Figure 9. During the experiment one slave motor was externally manipulated between 7 and 20 s, followed by dictating an equilibrium point trajectory by the master while the slave is kept fixed between 22 and 35 s, followed by a free moving slave tracking the equilibrium point as set by the master between 35 and 50 s. In the initial configuration, the motor controller budgets are empty, since the error energy is zero. That means that the motor controllers do not allow any positive power to flow from the motors to the mechanical system. However, it can be seen in Figure 9 that upon external manipulation of only one slave motor, the corresponding budget (Controller 1) increases due to the power outflow of the motor being negative, i.e., the motor absorbs power from the system. This means that the total energy in the system, which is the sum of the supervisor and motor controller budgets, is increased. When keeping the slave fixed while manipulating the master (in such a way that again only one slave motor would have to be actuated), energy from the supervisor does flow to the motor controller budget. Note that only that particular motor budget receives energy to perform a task, as properly calculated according to (15). This allocated energy is what has been calculated to be necessary in order for the slave to compensate the error energy in the system. When the master end effector returns to its initial position, the energy content in Controller 1 decreases and flows back to the supervisor, since that energy is not necessary anymore for decreasing the error energy. Note that the total energy in the system is constant. When releasing the slave and performing the same motion with the master as before, energy is allocated from the supervisor to the budget, and the total energy decreases due to dissipation. “Controllers” indicates the sum of the motor controller energy budgets, “Mechanism” indicates the kinetic energy of the slave, and “Virtual” indicates the virtual impedance controller energy.

#### 5.3.2. Traditional vs. energy-based

The supervisor and the motor controllers are both set at a sampling frequency of 100 Hz, and the back-and-forth motion, tracking the virtual equilibrium point with a free moving slave, and manipulating and suddenly releasing the slave were sequentially performed with both a traditional and the energy-based controller.

**Figure 10:**
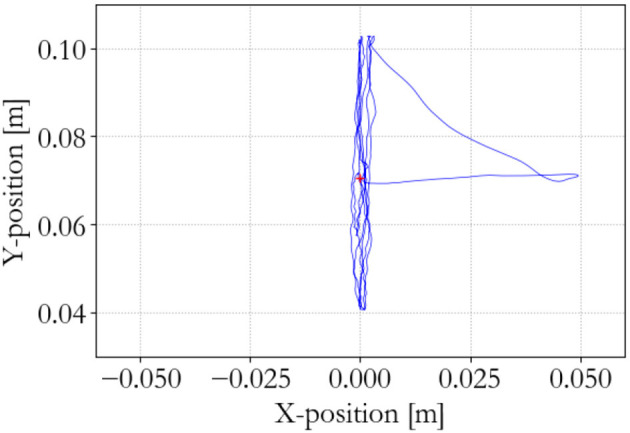
Master end effector position; traditional 100 Hz supervisor.

**Figure 11:**
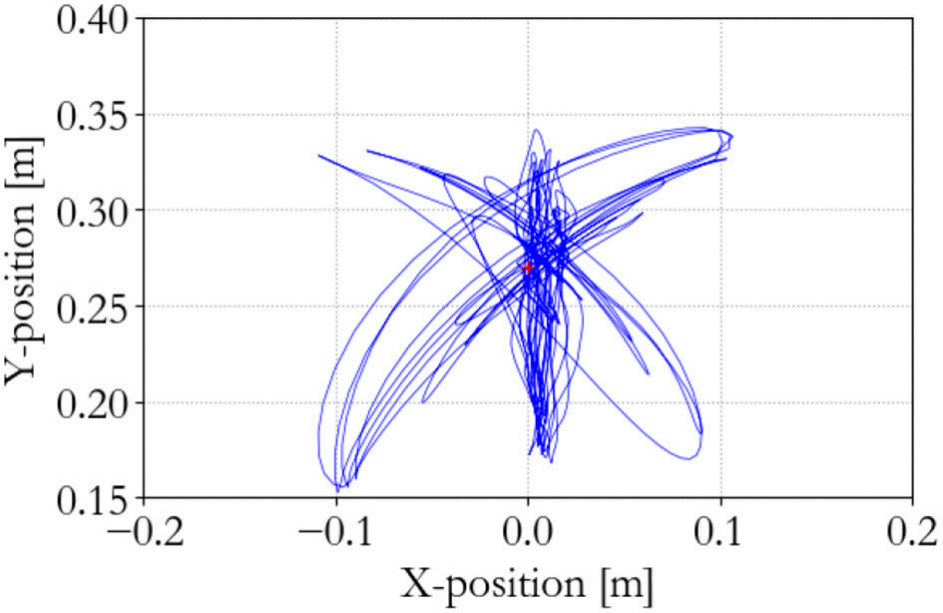
Slave end effector position; traditional 100 Hz supervisor.

**Figure 12:**
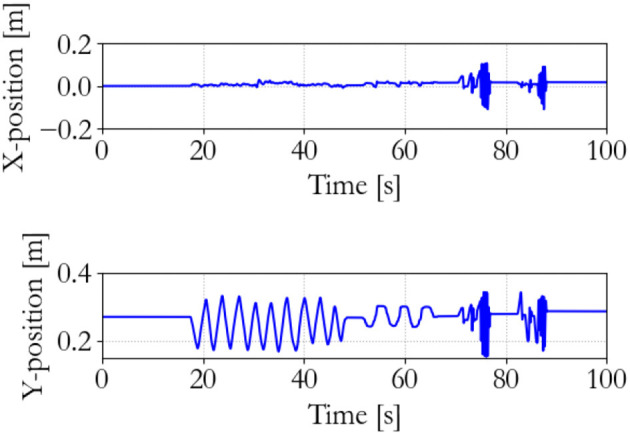
Slave end effector positions; traditional 100 Hz supervisor. Instability occurs at 75 s.

The performance of the traditional controller is shown in Figures 10–12. It can be seen that the discrete implementation of the traditional impedance controller at this sampling frequency is stable during back-and-forth motion and free space tracking of the equilibrium point. However, manipulating the slave and suddenly releasing it induces unstable oscillations in the system, which means that passivity has been lost.

**Figure 13:**
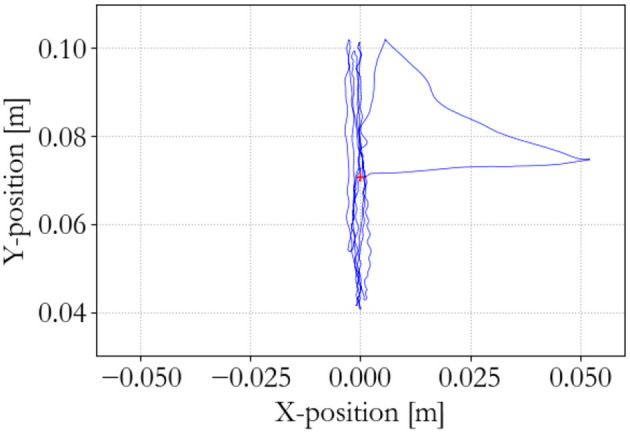
Master end effector position; energy-based 100 Hz supervisor.

**Figure 14:**
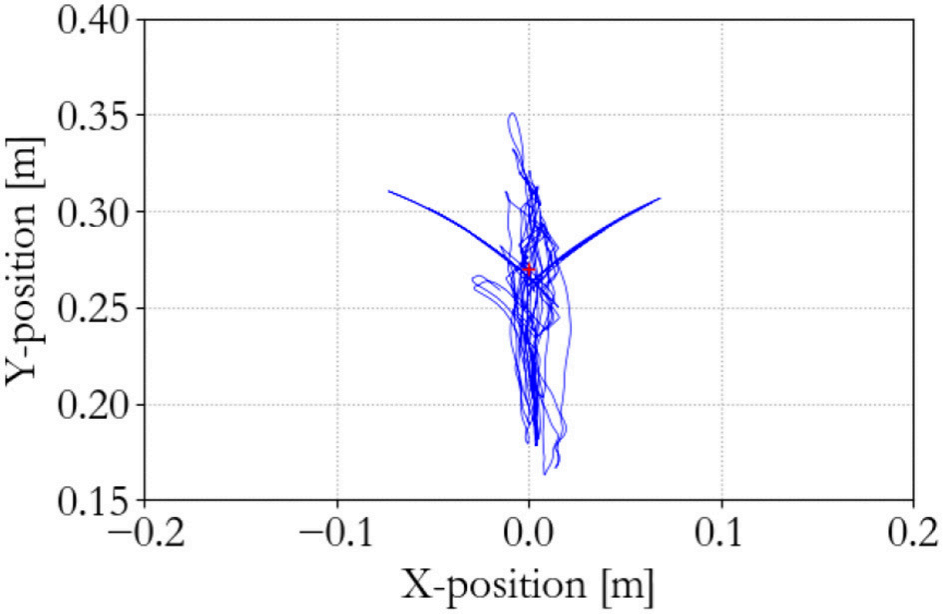
Slave end effector position; energy-based 100 Hz supervisor.

**Figure 15:**
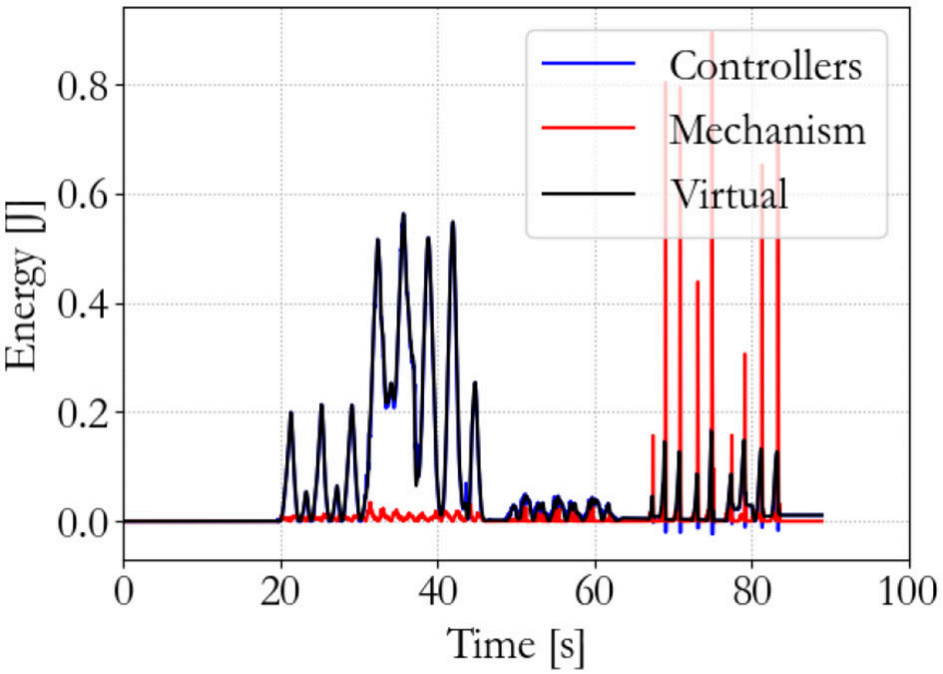
Slave energy levels; energy-based 100 Hz supervisor. No instability occurs.

The performance of the energy-based controller is seen in Figures 13–15, which is similar to the traditional controller. However, now all sequential experiments, including charging and suddenly releasing the slave, remain stable as opposed to the traditional controller.

The energy-based controller is aware of the energy that is injected into the system and can, therefore, ensure passivity and stability of the system. The traditional controller may violate passivity and allow so much energy injection in the system that it becomes unstable. The effectiveness of this paradigm has been clearly demonstrated in the previous experiments.

## 6. Discussion

The experiments shown here verify that the energy-based controller is able to keep the passivity of a system, as opposed to a traditional straight-forward implementation. This shows that the proposed approach is a viable method to control robotic architectures to ensure stability while obtaining adequate performance. The energy-based controller will not create instability in the system due to the awareness of the amount of energy that has been injected in the system. Also, performance is not considerably decreased due to the appropriate estimation of the actuation controller budgets in this situation. The traditional controller is unaware of the injected energy and will become unstable and, therefore, unusable in certain situations. A complete and persistent communication loss between the supervisor and the actuation controllers will also not cause instability, since the actuators are aware of the injected energy. It is noted that this approach is independent of the chosen control algorithm in the high-level supervisor. Here, an impedance controller is implemented and is mainly used for interaction, while it was briefly shown that is also works for a position regulation task without interaction. If an energy error can be defined between the desired system states and the actual system states, and the two conditions as mentioned in section 3 are fulfilled, energy budget allocations can be estimated and passivity can be successfully implemented using this approach.

The experiments performed here were done with a controller that is based on a model of the system. Energy budget estimations may deteriorate when the controller approaches the model validity boundaries, which can cause the energy estimation to be too generous or conservative. This mainly affects the performance of the system, since the energy budgeting is still limited, so unbounded growth of system states cannot occur. Moreover, the proposed method assumes perfect measurements of the actuation torque and velocity or position, while they may be different from their setpoint or may be quantized to implement in a digital controller. The effects of these issues have been partially investigated in Stramigioli et al. (2005), and should be further researched in the future.

An important aspect to realize, which is also not treated in this work is that energy budgets will be communicated over a network architecture as packets. This means that the total energy is not only the sum of the energy budgets in the supervisor and individual controllers, but also the “traveling” energy in the network. To accurately keep passivity of the system, this aspect should be taken into account.

## 7. Conclusion

This paper proposed a unified energy-based modeling and energy-aware control paradigm for robotic systems. It presented an energy-based modeling framework that is applicable to any robotic system, in which the energy transfer between subsystems is made explicit. It considers the separation of a high-level, and possibly relatively slow, supervisory control system, and the lower-level, and likely relatively fast, actuation controllers. By implementing energy-awareness on the actuation controllers, stability can be guaranteed through passivity, even under large time delays or communication losses. The actuation controllers can expend an energy budget to fulfill a certain task, and are incapable of injecting more energy if the budget has depleted. An approach to allocate these budgets was proposed, while a strategy was presented to follow when a budget has depleted. Experiments validated that the proposed method is capable of stably controlling an interacting mechanical five bar linkage system, as opposed to a traditional controller which destabilizes the system quickly.

## Author contributions

SG was involved in the development of the conceptual framework that is presented in the manuscript and the implementation on a physical system, and has taken the lead in writing the manuscript and shaping it into its current form. SS was involved in the preliminary work related to this research topic, and contributed to the conceptualization of the framework presented in the manuscript. GF was involved in earlier work which has formed the substantial basis for the presented work in this manuscript, and was involved in furthering the research topic. Moreover, GF has provided help with proof reading. Also, SS has provided help with proof reading and shaping the manuscript into its final form.

### Conflict of interest statement

The authors declare that the research was conducted in the absence of any commercial or financial relationships that could be construed as a potential conflict of interest.

## Footnotes

^1^The reverse correspondence may hold in some situations, for instance in case of generalized bond graph models. A treatment of various modeling decisions, however, is beyond the scope of this work.

## References

[B1] Alami R. Albu-Schaeffer A. Bicchi A. Bischoff R. Chatila R. De Luca A. (2006). Safe and dependable physical human-robot interaction in anthropic domains: state of the art and challenges, in 2006 IEEE/RSJ International Conference on Intelligent Robots and Systems (Beijing), 1–16.

[B2] Controllab Products B.V (2016). 20-sim.

[B3] Duindam V. Macchelli A. Stramigioli S. Bruyninckx H. (eds.). (2009). Modeling and Control of Complex Physical Systems: The Port-Hamiltonian Approach. Berlin; Heidelberg: Springer Science & Business Media.

[B4] Folkertsma G. A. Groothuis S. S. Stramigioli S. (2018). Safety and guaranteed stability through embedded energy-aware actuators, in IEEE International Conference on Robotics and Automation (Brisbane, QLD).

[B5] Greenwald R. M. Gwin J. T. Chu J. J. Crisco J. J. (2008). Head impact severity measures for evaluating mild traumatic brain injury risk exposure. Neurosurgery 62, 789–798. 10.1227/01.neu.0000318162.67472.ad 18496184 PMC2790598

[B6] Groothuis S. S. Stramigioli S. Carloni R. (2013). Lending a helping hand: toward novel assistive robotic arms. IEEE Robot. Automat. Mag. 20, 20–29. 10.1109/MRA.2012.2225473

[B7] Lee D. Huang K. (2010). Passive-set-position-modulation framework for interactive robotic systems. IEEE Trans. Robot. 26, 354–369. 10.1109/TRO.2010.2041877

[B8] Newman J. A. Shewchenko N. Welbourne E. (2000). A proposed new biomechanical head injury assessment function - the maximum power index. Stapp Car Crash J. 44, 215–247. 17458729 10.4271/2000-01-SC16

[B9] Ortega R. van der Schaft A. Castaños F. Astolfi A. (2008). Control by interconnection and standard passivity-based control of port-hamiltonian systems. IEEE Trans. Automat. Contr. 53, 2527–2542. 10.1109/TAC.2008.2006930

[B10] Purves D. Augustine G. J. Fitzpatrick D. Hall W. C. LaMantia A.-S. McNamara J. O. (eds.) (2012). Neuroscience, 5 Edn Sunderland: Oxford University Press.

[B11] Raiola G. Cardenas C. A. Tadele T. S. de Vries T. J. Stramigioli S. (2018). Development of a safety-aware intrinsically passive controller for collaborative robots. IEEE Robot. Automat. Lett. 3, 1237–1244, 10.1109/LRA.2018.2795639

[B12] Robotics Mechatronics (2018). RaMStix FPGA Board Documentation. Available online at: https://www.ram.ewi.utwente.nl/ecssoftware/ramstix/docs/index.html

[B13] Ryu J.-H. Kwon D.-S. Hannaford B. (2004). Stable teleoperation with time-domain passivity control. IEEE Trans. Robot. Automat. 20, 365–373. 10.1109/TRA.2004.824689

[B14] Ryu J.-H. Preusche C. Hannaford B. Hirzinger G. (2005). Time domain passivity control with reference energy following. IEEE Trans. Contr. Syst. Technol. 13, 737–742. 10.1109/TCST.2005.847336

[B15] Schindlbeck C. Haddadin S. (2015). Unified passivity-based cartesian force/impedance control for rigid and flexible joint robots via task-energy tanks, in 2015 IEEE International Conference on Robotics and Automation (ICRA) (Seattle), 440–447.

[B16] SoftPro (2017). SoftPro Available online at : http://softpro.eu

[B17] Stramigioli S. (2001). Modeling and IPC Control of Interactive Mechanical Systems: A Coordinate-free Approach. Number 266 in Lecture Notes in Control and Information Sciences. London, UK: Springer Verlag London Ltd.

[B18] Stramigioli S. (2015). Energy-aware robotics, in Mathematical Control Theory I: Nonlinear and Hybrid Control Systems, Vol. 461 of Lecture Notes in Control and Information Sciences, eds Camlibel M. K. Julius A. A. Pasumarthy R. Scherpen J. M. (Cham: Springer International Publishing), 37–50.

[B19] Stramigioli S. Secchi C. Van der Schaft A. Fantuzzi C. (2005). Sampled data systems passivity and discrete port-hamiltonian systems. IEEE Trans. Robot. 21, 574–587. 10.1109/TRO.2004.842330

[B20] Tadele T. S. de Vries T. J. Stramigioli S. (2014). Combining energy and power based safety metrics in controller design for domestic robots, in IEEE International Conference on Robotics and Automation (Hong Kong).

[B21] van der Schaft A. (1999). L2-Gain and Passivity in Nonlinear Control. Berlin; Heidelberg: Springer-Verlag New York, Inc.

[B22] van der Schaft A. (2006). Port-hamiltonian systems: an introductory survey, in Proceedings of the International Congress of Mathematicians (Madrid).

[B23] van der Schaft A. Cervera J. (2002). Composition of Dirac Structures and Control of Port-Hamiltonian Systems, in Proceedings of the 15th International Symposium on the Mathematical Theory of Networks and Systems (South Bend: University of Notre Dame).

[B24] Willems J. C. (1972). Dissipative dynamical systems part I: General theory. Arch. Ration. Mech. Anal. 45, 321–351.

[B25] Yavetz I. (1995). From Obscurity to Enigma: The Work of Oliver Heaviside, 1872–1889. Basel: Birkhäuser Verlag.

